# Case report: Scleromalacia caused by rheumatoid arthritis combined with high intraocular pressure, first discovered due to ocular trauma

**DOI:** 10.3389/fmed.2024.1504807

**Published:** 2025-01-30

**Authors:** Yanyan Zhang, Yanyan Wang, Ruihao Xu, Tianyu Wang, Yuhao Zhang, Jinghai Mao, Quanyong Yi

**Affiliations:** ^1^Ningbo Eye Hospital, Wenzhou Medical University, Ningbo, Zhejiang, China; ^2^The First Affiliated Hospital, Jiangxi Medical College, Nanchang University, Nanchang, Jiangxi, China

**Keywords:** bluish sclera, scleromalacia, rheumatoid arthritis, ocular trauma, ocular hypertension

## Abstract

A 53-year-old man with rheumatoid arthritis was first diagnosed with scleromalacia at the ophthalmology clinic after experiencing ocular trauma. The patient presented with decreased vision and abnormalities were subsequently found in his other eye. An ophthalmologist performed emergency debridement and suturing surgery on the ruptured right eyeball, discovering that the sclera was thinned to only 1/5 to 1/3 of its normal thickness. After the operation, the ophthalmologist examined the patient’s right eye and noted similar bluish scleral changes along with elevated intraocular pressure. In this case, we report a relatively rare instance of scleromalacia combined with ocular hypertension, which was definitively diagnosed following emergency ophthalmic surgery. The patient exhibited extremely thin and bluish sclera in both eyes. This case once again underscores the importance of clinicians paying close attention to the impact of systemic autoimmune diseases on ocular health.

## Introduction

Scleromalacia is a rare but serious complication of long-standing rheumatoid arthritis (1–3). It often presents with minimal redness, pain, or other discomfort (4). The thinning and fragility of the sclera caused by the rheumatoid arthritis significantly increase the risk of ocular trauma. Additionally, scleromalacia may occur in conjunction with intraocular hypertension or glaucoma, reminding us of the need to monitor for occult glaucoma (5, 6).

## Case report

**Figure 1:**
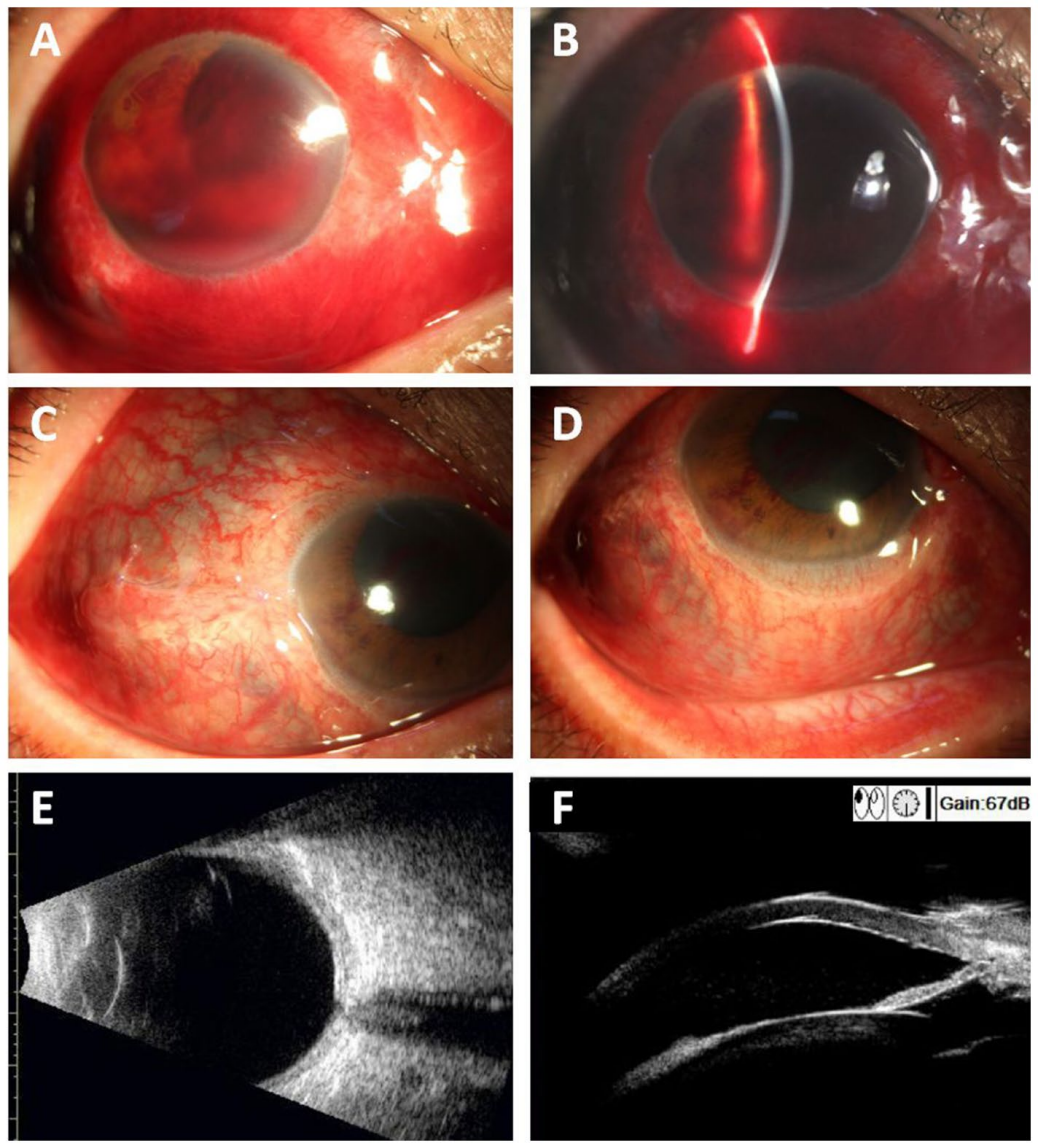
Preoperative photographs of the injured right eye show visible bluish discoloration and scleral thinning. **(A,B)** Preoperative ocular photographs of the injured right eye (Yellow circles show areas of scleral thinning). **(C,D)** Postoperative ocular photographs of the right eye 3 weeks after surgery. The yellow arrow indicates the thin sclera with visible subconjunctival uvea. **(E)** Ocular B-ultrasonography showed mild vitreous opacity in the right eye and slight thickening of the eyeball wall. **(F)** UBM examination showed an open anterior chamber angle in the right eye.

A 53-year-old man with a history of rheumatoid arthritis, who had not received any anti-rheumatic treatment, presented to the ophthalmology clinic for an emergency operation due to decreased vision following a bottle striking his right eye. His best corrected visual acuity (BCVA) was light perception in the right eye and 20/30 in the left eye (Figures 1A,B). The right eye injury was classified as Grade 4 based on visual acuity at the time of presentation, and the injury extending beyond the anterior 5 mm of the sclera was categorized as zone III (7). An ophthalmologist performed emergency debridement and suturing surgery for the ruptured right eyeball. Preoperative slit-lamp examination showed scattered black choroidal-like reflections under the conjunctiva (Figures 1C,D). During surgery, the sclera was found to be thinned to just 1/5–1/3 of normal thickness. The upper nasal and temporal regions were ruptured due to the overly thin sclera and trauma, and even new scleral holes were formed in the thinnest areas by the microsuture needles. Postoperative ocular B ultrasonography showed mild vitreous opacity and slight thickening of the eyeball wall in the right eye (Figure 1E). UBM examination showed an open anterior chamber angle in the right eye (Figure 1F).

**Figure 2:**
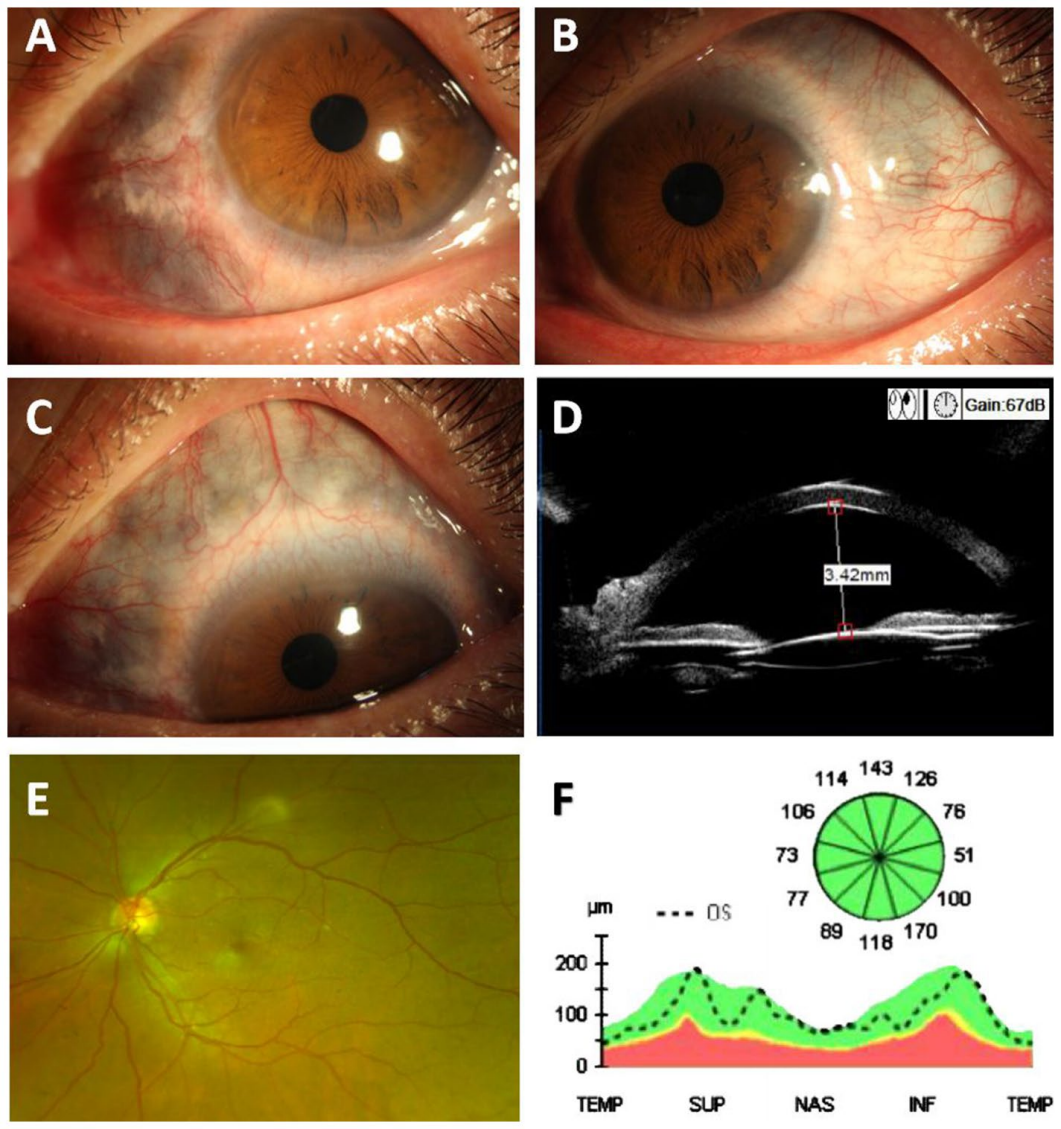
Photographs of the left eye show visible bluish discoloration and scleral thinning. **(A–C)** Ocular photographs of the left eye (Yellow circles show areas of scleral thinning). **(D)** UBM examination showed an open anterior chamber angle in the left eye. **(E)** Widefield color fundus photography of the left eye. **(F)** OCT examination of the optic nerve thickness in the left eye.

The patient had suffered from rheumatoid arthritis for 15 years. Two years prior, cataract surgery on the left eye was performed without complications, and no indications of high intraocular pressure or scleral thinning were noted during routine follow-up. Cataract phacoemulsification and intraocular lens implantation were completed successfully. He was unaware of his long-standing bluish discoloration of the eyes (Figures 2A–C).

He denied any history of fractures or hearing loss and had no joint pain or painful red ocular symptoms before the injury, but synovial hypertrophy was observed in the second and third metacarpophalangeal joints bilaterally. Three weeks after this surgery, his visual acuity in the right eye remained “count fingers” at close distance, and the fundus was unclear due to traumatic cataract. Intraocular pressure was measured with TR (right eye pressure) recorded as 13 mmHg and TL (left eye pressure) as 28.3 mmHg. Despite trauma, the anterior chamber angles in both eyes remained open, as confirmed by UBM examination (Figures 1F, 2D). No significant abnormalities were found in the fundus of the left eye (Figure 2E). The optic nerve thickness was normal (Figure 2F), and perimetry results were unreliable due to poor patient cooperation.

## Discussion

In this case, physical examination revealed a bluish hue in the sclerae of both eyes (yellow arrows). Rheumatoid arthritis is one of the most common causes of scleritis, with chronic inflammation leading to thinning of the sclera, which appears blue due to reflection of the underlying choroidal pigment (8). Clinicians should be aware that long-standing rheumatoid arthritis can result in scleral thinning, giving the eyes a bluish appearance as the underlying uvea becomes visible. Extremely thin sclera is highly susceptible to trauma, even from lightweight impacts. Although scleromalacia may occur alongside intraocular hypertension or glaucoma, spontaneous perforation is rare. Complications such as ocular hypertension can predispose these patients to occult glaucoma. In this case, the patient’s thin sclera was ruptured by an empty plastic bottle. In emergency cases, direct scleral suturing was performed due to the urgency of the situation. However, literature suggests that allogeneic sclera or amniotic membrane grafts could provide better structural support and long-term prognosis for scleral defects, warranting consideration in similar future cases (9, 10). During surgery, the sclera was noted to be extremely thin, with elevated intraocular pressure, normal nerve fiber layer thickness, and poor perimetry results due to lack of coordination. A thorough history and examination are essential for distinguishing episcleritis from other causes of blue sclera, as well as for diagnosing, treating, and monitoring this condition. This case also underscores the importance of protecting the patient’s uninjured eye.

Scleral thinning can result from a range of etiologies, including congenital, degenerative, immunological, and infectious causes. Scleromalacia differs from other scleral diseases in its presentation and pathology (8). Congenital conditions such as osteogenesis imperfecta and Ehlers-Danlos syndrome are characterized by collagen abnormalities, leading to structural weakness of the sclera and a bluish discoloration due to choroidal visibility. Degenerative processes, exemplified by high myopia, involve progressive elongation of the globe, which may thin the sclera, particularly in the posterior pole. Immunological diseases such as rheumatoid arthritis (RA), as described in this case, induce chronic inflammation mediated by cytokines (e.g., TNF-*α*, IL-6), degrading the extracellular matrix of the sclera and increasing susceptibility to trauma (5). Infectious scleritis, often caused by bacterial, fungal, or parasitic pathogens, leads to localized scleral thinning, sometimes with abscess formation (11). Scleromalacia differs from episcleritis in its lack of pain and vascular congestion. Necrotizing scleritis, often associated with significant pain, shows potential for perforation, while infectious scleritis is usually localized with abscess formation. The hallmark bluish hue in scleromalacia results from extreme thinning of the sclera, exposing the underlying uvea, which is absent in these other conditions.

Accurate differentiation among these causes requires detailed clinical evaluation. For example, osteogenesis imperfecta may involve a history of fractures, hearing loss, and other connective tissue symptoms, while Ehlers-Danlos syndrome often presents with systemic joint hypermobility. Immunological causes like RA are typically associated with synovial inflammation and systemic autoimmunity, as highlighted in our patient’s 15-year history. Infectious causes often exhibit localized abscesses, tenderness, and signs of systemic infection. Comprehensive management, including systemic control and surgical interventions such as scleral grafting, should be tailored to the underlying etiology (12).

Management of scleral thinning involves systemic treatment with immunosuppressive agents, alongside surgical interventions like scleral grafting or amniotic membrane transplantation for structural support. Timely intervention and systemic disease control significantly improve prognosis.

Similar cases have documented the link between rheumatoid arthritis and ocular complications. For example, Dean et al. highlighted secondary glaucoma due to chronic scleritis (5), while Kocak et al. reported neovascular glaucoma in the presence of scleromalacia (6). Elevated IOP in rheumatoid arthritis patients with scleromalacia can arise from various mechanisms (13): Chronic inflammation may lead to trabecular meshwork dysfunction, impairing aqueous humor outflow. Long-term oral hormonal drugs such as dexamethasone may lead to elevated IOP. Scarring or fibrosis of the outflow pathways caused by pro-inflammatory cytokines further exacerbates IOP elevation. In some cases, neovascularization, as a response to chronic ischemia or inflammation, can contribute to angle closure and secondary glaucoma (14).

This case highlights the importance of prioritizing regular eye examinations and protective measures for patients with systemic diseases like rheumatoid arthritis to minimize the risk of trauma and inflammation-related damage. Additionally, managing the systemic disease is crucial, emphasizing the need for collaboration between ophthalmology and rheumatology. Research indicates that ocular surface complications can precede or exacerbate systemic diagnoses (11, 15). By recognizing ocular manifestations of systemic rheumatic diseases, it may be possible to prevent or at least delay many long-term sequelae.

### Differential diagnoses

1. Medication-induced scleral discoloration.
2. Ocular melanoma.
3. Osteogenesis imperfecta.
4. Van Der Heave syndrome.
5. Ehlers-Danlos syndrome.

## Data Availability

The original contributions presented in the study are included in the article/supplementary material, further inquiries can be directed to the corresponding authors.

## References

[1] Alhassan E. Blue sclerae in rheumatoid arthritis. JCR. J Clin Rheumatol. (2023) 29:e134. doi: 10.1097/RHU.0000000000001994, PMID: 37370216

[2] de Figueiredo LQ de Andrade Lopes FO Franco AS Giardini HAM Guedes LKN Bonfiglioli KR. Scleromalacia perforans as an early manifestation of late-onset rheumatoid arthritis: a case-based review. Rheumatol Int. (2024) 44:1165–73. doi: 10.1007/s00296-023-05494-0, PMID: 37925382

[3] Wolf MD Lichter PR Ragsdale CG. Prognostic factors in the uveitis of juvenile rheumatoid arthritis. Ophthalmology. (1987) 94:1242–8. doi: 10.1016/s0161-6420(87)80007-6, PMID: 3684202

[4] Reddy AK Kolfenbach JR Palestine AG. Ocular manifestations of rheumatoid arthritis. Curr Opin Ophthalmol. (2022) 33:551–6. doi: 10.1097/ICU.0000000000000890, PMID: 36165413

[5] Dean WH Turner SA McNaught AI. Secondary glaucoma due to chronic scleritis: trabeculectomy in scleromalacia: a case report. Eye (Lond). (2014) 28:104–6. doi: 10.1038/eye.2013.215, PMID: 24232311 PMC3890753

[6] Kocak AA Ilhan C Citirik M. Unusual association of inverse retinitis Pigmentosa, scleromalacia, and neovascular glaucoma. Turk J Ophthalmol. (2020) 50:110–4. doi: 10.4274/tjo.galenos.2020.07573, PMID: 32367703 PMC7204902

[7] Dogramaci M Erdur SK Senturk F. Standardized classification of mechanical ocular injuries: efficacy and shortfalls. Beyoglu Eye J. (2021) 6:236–42. doi: 10.14744/bej.2021.01488, PMID: 35005522 PMC8697050

[8] Turk MA Hayworth JL Nevskaya T Pope JE. Ocular manifestations in rheumatoid arthritis, connective tissue disease, and vasculitis: a systematic review and metaanalysis. J Rheumatol. (2021) 48:25–34. doi: 10.3899/jrheum.190768, PMID: 32358156

[9] Carriel V Vizcaíno-López G Chato-Astrain J Durand-Herrera D Alaminos M Campos A. Scleral surgical repair through the use of nanostructured fibrin/agarose-based films in rabbits. Exp Eye Res. (2019) 186:107717. doi: 10.1016/j.exer.2019.107717, PMID: 31265829

[10] Kang M Feng H Xu X Wang Z Zhang Y Chen K. Report of endophthalmitis caused by Paradictyoarthrinium diffractum after plant trauma: a case involving left enucleation. Int J Infect Dis. (2024) 146:107117. doi: 10.1016/j.ijid.2024.107117, PMID: 38801967

[11] Hysa E Cutolo CA Gotelli E Paolino S Cimmino MA Pacini G. Ocular microvascular damage in autoimmune rheumatic diseases: the pathophysiological role of the immune system. Autoimmun Rev. (2021) 20:102796. doi: 10.1016/j.autrev.2021.102796, PMID: 33722750

[12] Turgut F Dingerkus V Tappeiner C Becker M. Diagnostisches und therapeutisches management der episkleritis und skleritis. Klin Monatsbl Augenheilkd. (2023) 240:725–38. doi: 10.1055/a-2022-0689, PMID: 36827997

[13] Geyer O Levo Y. Glaucoma is an autoimmune disease. Autoimmun Rev. (2020) 19:102535. doi: 10.1016/j.autrev.2020.102535, PMID: 32234407

[14] Samuelson TW Huang MJ Larsen CL Sheybani A Levin A Ertel M. Extreme intraocular pressure and steroid-dependent iritis. J Cataract Refract Surg. (2023) 49:108–13. doi: 10.1097/j.jcrs.0000000000001104, PMID: 36573765

[15] Tong L Thumboo J Tan YK Wong TY Albani S. The eye: a window of opportunity in rheumatoid arthritis? Nat Rev Rheumatol. (2014) 10:552–60. doi: 10.1038/nrrheum.2014.85, PMID: 24914693

